# Mortality of Philadelphia for 1862

**Published:** 1863-08

**Authors:** 


MORTALITY OF PHILADELPHIA FOR 1862. REPORT ON METEOROLOGY AND EPIDEMICS. Read before the College of Physicians of Philadelphia, Feb. 4th, 1863. By WILSON JEWELL, M.D., President of the Medical Society of Pennsylvania, &c., &c.

This is a very interesting and valuable statistical paper of forty printed pages, by one who cultivates zealously and successfully the field of hygiene. A similar report should be published annually in relation to every city and county in the whole country.

